# The effect of case management and vector-control interventions on space–time patterns of malaria incidence in Uganda

**DOI:** 10.1186/s12936-018-2312-7

**Published:** 2018-04-12

**Authors:** Julius Ssempiira, John Kissa, Betty Nambuusi, Carol Kyozira, Damian Rutazaana, Eddie Mukooyo, Jimmy Opigo, Fredrick Makumbi, Simon Kasasa, Penelope Vounatsou

**Affiliations:** 1 Swiss Tropical and Public Health Institute, Socinstrasse 57, 4051 Basel, Switzerland; 2 University of Basel, Petersplatz 1, 4001 Basel, Switzerland; 3 Makerere University School of Public Health, New Mulago Hospital Complex, P.O Box 7072, Kampala, Uganda; 4 Uganda Ministry of Health, Plot 6 Lourdel Road, P.O. Box 7272, Nakasero, Kampala, Uganda

**Keywords:** Artemisinin-based combination therapy (ACT), Bayesian inference, Conditional autoregressive (CAR) model, District Health Information Software System version 2 (DHIS2), Malaria interventions, Insecticide treated nets (ITN), Negative binomial

## Abstract

**Background:**

Electronic reporting of routine health facility data in Uganda began with the adoption of the District Health Information Software System version 2 (DHIS2) in 2011. This has improved health facility reporting and overall data quality. In this study, the effects of case management with artemisinin-based combination therapy (ACT) and vector control interventions on space–time patterns of disease incidence were determined using DHIS2 data reported during 2013–2016.

**Methods:**

Bayesian spatio-temporal negative binomial models were fitted on district-aggregated monthly malaria cases, reported by two age groups, defined by a cut-off age of 5 years. The effects of interventions were adjusted for socio-economic and climatic factors. Spatial and temporal correlations were taken into account by assuming a conditional autoregressive and a first-order autoregressive AR(1) process on district and monthly specific random effects, respectively. Fourier trigonometric functions were incorporated in the models to take into account seasonal fluctuations in malaria transmission.

**Results:**

The temporal variation in incidence was similar in both age groups and depicted a steady decline up to February 2014, followed by an increase from March 2015 onwards. The trends were characterized by a strong bi-annual seasonal pattern with two peaks during May–July and September-December. Average monthly incidence in children < 5 years declined from 74.7 cases (95% CI 72.4–77.1) in 2013 to 49.4 (95% CI 42.9–55.8) per 1000 in 2015 and followed by an increase in 2016 of up to 51.3 (95% CI 42.9–55.8). In individuals ≥ 5 years, a decline in incidence from 2013 to 2015 was followed by an increase in 2016. A 100% increase in insecticide-treated nets (ITN) coverage was associated with a decline in incidence by 44% (95% BCI 28–59%). Similarly, a 100% increase in ACT coverage reduces incidence by 28% (95% BCI 11–45%) and 25% (95% BCI 20–28%) in children < 5 years and individuals ≥ 5 years, respectively. The ITN effect was not statistically important in older individuals. The space–time patterns of malaria incidence in children < 5 are similar to those of parasitaemia risk predicted from the malaria indicator survey of 2014–15.

**Conclusion:**

The decline in malaria incidence highlights the effectiveness of vector-control interventions and case management with ACT in Uganda. This calls for optimizing and sustaining interventions to achieve universal coverage and curb reverses in malaria decline.

**Electronic supplementary material:**

The online version of this article (10.1186/s12936-018-2312-7) contains supplementary material, which is available to authorized users.

## Background

The launch of the Roll Back Malaria (RBM) programme and the Global Fund to Fight AIDS, Tuberculosis and Malaria marked the first serious international efforts to control and prevent malaria in sub-Saharan Africa (SSA), since the global malaria eradication programme was abandoned in the 1970s [1]. These efforts have accelerated the scale-up of vector control interventions and case management with artemisinin-based combination therapy (ACT) in endemic countries leading to a significant decline in malaria morbidity and mortality [2]. In spite of this success, malaria still remains a public health problem in the majority of countries in SSA with the heaviest burden borne in children less than 5 years old [3].

In Uganda, the scaling-up of interventions resulted in the decline of malaria parasitaemia risk during 2009–2015 [4, 5], but nonetheless, the country still ranks among the top six high burdened in the world [6]. The Uganda Health Management Information System (HMIS) was established in the early 1990s to facilitate reporting of routine health facility data to the Ministry of Health (MoH) [7]. The system has since undergone several revisions and multiple technological upgrades to strengthen health facility and district-based reporting and improve reporting of routine health facility data. The most crucial improvement was the adoption of the District Health Information Software System version 2 (DHIS2) in 2011 which facilitated the transition from a paper-based reporting and storage to an electronic web-based system in 2011.

To ensure a fast and effective roll-out process, the Ministry of Health (MoH) with support from international partners conducted 35 regional training workshops during January 2011–January 2012 for all district records assistants, Biostatisticians, health officers and HMIS focal persons. By July 2012, all districts were using DHIS2 online and reporting monthly HMIS data, thanks to the strong IT capacity of the MoH staff, technical and financial support from CDC and USAID. In spite of some challenges in the beginning, such as internet connectivity issues and limited workforce there was a great improvement in health reporting after the introduction of DHIS2 in 2012/13 compared to the period before 2012. Completeness and timeliness of outpatient reporting increased from 36 and 22% in 2011/12 to 85 and 77% in 2012/13, respectively. Also, most child-related health coverage indicators increased from about 50% in 2011/12 to over 80% in 2012/13 [8].

However, routine health facility data utilization in Uganda remains low and disease burden estimation relies mainly on population-based surveys such as the Demographic Health Survey (DHS) and Malaria Indicator Survey (MIS) [9]. MIS are conducted periodically every 5 years to estimate malaria parasite prevalence in children less than 5 years [10, 11]. The DHIS2 data, on the other hand, provides an opportunity to investigate inter and intra-annual variation of malaria risk in individuals for all age groups presenting with malaria to health facilities. The adoption of ‘Test and Treat’ campaign by MoH has greatly improved the number of health facility malaria cases confirmed by the rapid diagnostic tests (RDTs) [6]. This data can provide a wealth of information for monitoring and evaluation of malaria programming activities to support evidence-based decision making.

Routine health facility data are spatially and temporally correlated due to common exposures in proximal areas and time points. Bayesian Conditional Autoregressive (CAR) models adjust for spatial correlation in district-level incidence and smooth disease rates to highlight the spatial pattern of the true burden and produce unbiased parameter estimates [12]. Bayesian space–time CAR models have been applied to analyse malaria cases routinely collected from health facilities in Namibia [13], Venezuela [14], Mozambique [15], Malawi [16], Zimbabwe [17], China [18] and in South Africa [19]. These studies investigated effects of environmental and socio-economic factors on inter and intra annual variation of malaria incidence.

In this work, Bayesian negative binomial CAR models were fitted on district-aggregated monthly malaria cases reported in the DHIS2 during 2013–2016 to estimate the effects of malaria interventions on the spatio-temporal patterns of the disease incidence in Uganda in children less than 5 years and individuals of 5 years and above. The models were adjusted for climatic and socio-economic factors. The results provide important information to National Malaria Control Programme (NMCP) for evaluating progress and for planning the timing and priority areas to allocate malaria interventions.

## Methods

### Settings

Uganda is located in East Africa on a large plateau in the great lakes region. Its altitude varies between 1300 and 1500 m above sea level and the mean annual temperature ranges from 16 to 30 °C. It has a diverse vegetation, mainly comprising of tropical rainforests in the South, wooded savanna in Central, and semi-arid in the North and North East regions. There are two rainy seasons; the first during March–May and the second from August to November. The population is 37 million, of which 18% are children < 5 years [20]. The country is divided into 112 districts and covers an area of 241,039 square kilometres.

Malaria transmission rates are among the highest in the world [21]. Transmission is stable in 95% of the country. Low and unstable transmission is mainly present in the highland areas. Malaria is responsible for 33% of outpatient visits and 30% of hospitalized cases. *Anopheles gambiae* sensu lato (s.l.) is the dominant vector species followed by *Anopheles funestus,* which is commonly found in areas having permanent water bodies with emergent vegetation. These two vectors are strongly endophilic and endophagic that is, feeding indoors and resting on walls after feeding, which makes vector control approaches effective. Health facilities in Uganda are classified and graded according to their service scope and size of the population they serve in the following (descending) order; hospitals, Health Center (HC) IVs, HCIIIs and HCIIs. At the time of conducting this study, there were 5418 health facilities; 160 hospitals, 197 HCIVs, 1289 HCIIIs and 3772 HCIIs.

### Data sources

#### Malaria cases

Data on confirmed malaria cases by RDT was extracted from the DHIS2 covering the period of January 2013 to December 2016. The data were reported by two age groups: children < 5 years and individuals ≥ 5 years. Malaria incidence in each age group was estimated by dividing the district aggregated malaria cases by the district age group-specific population. The populations for 2013, 2015 and 2016 were estimated using the national housing and population census of 2014 adjusted for the annual population growth rate [20].

#### Malaria interventions, socio-economic and climate data

Malaria interventions data that is, ITN and case management with Artemisinin Combination Therapies (ACT) were obtained from the MIS 2014–15 [10]. Indoor residual spraying (IRS) was not included in the analysis because of its sparse distribution in the majority of the districts owing to the targeted implementation strategy used in its deployment [6]. Six ITN coverage indicators were defined from the MIS 2014–15; corresponding to three ownership and three use indicators defined by Roll Back Malaria (RBM) namely; proportion of households with at least one ITN, proportion of households with at least one ITN for every two people, proportion of population with access to an ITN in their household, proportion of the population that slept under an ITN the previous night, proportion of children under 5 years old who slept under an ITN the previous night, proportion of existing ITNs used the previous night. Also, the wealth score computed from household possessions captured in the MIS 2014–15 questionnaires was used as a socio-economic proxy. A wealth index of five quintiles was generated from the score based on the data distribution following the DHS methodology [22]. Environmental and climatic data were downloaded from remote sensing sources during October 2012–August 2016. Day and night land surface temperature (LST), Normalized Difference Vegetation Index (NDVI), and land cover were extracted from Moderate Resolution Imaging Spectroradiometer (MODIS) at a spatial resolution of 1 × 1 km^2^ and temporal resolution of 8, 16 days and annually, respectively. Dekadal rainfall data was obtained from the US early warning and environmental monitoring system at 8 × 8 km^2^ resolution. Altitude was extracted from the shuttle radar topographic mission using the digital elevation model. The ESRI’s ArcGIS 10.2.1 was used to estimate distances between major water bodies and district centroids (http://www.esri.com/). Details of climatic data processing have been explained in Additional file 1.

### Statistical analysis

The analysis was carried out separately for each age group, i.e. children < 5 and individuals ≥ 5 years old. Time series plots were employed to describe inter and intra-annual variation of malaria incidence and temporal variation of environmental/climatic factors during the study period.

Biological considerations of the malaria transmission cycle suggest that there is an elapsing period between climatic suitability for malaria transmission and occurrence of cases, which is related to climatic effects on the duration of the sporogony cycle i.e. the development of the parasite within the mosquito [23]. We took this into account by creating lagged variables for the time-varying climatic predictors (i.e. rainfall, NDVI, day LST and night LST). In particular, three analysis variables were constructed for each climatic factor by averaging its values over the following periods: the current and the previous month (lag1), the current and the two previous months (lag2) and the current and the three previous months (lag3). Categorical variables were generated based on tertiles of the variables’ distributions since the relationship between malaria and environmental predictors is not always linear [24].

Bayesian spatio-temporal negative binomial models were fitted on the incidence data. Heterogeneity in incidence was taken into account via year-specific, spatially structured and unstructured random effects modeled at district level via CAR and Gaussian exchangeable prior distributions, respectively [25]. The nested space–time structure allowed the geographical variation of malaria to vary from year to year. Furthermore, temporal correlation across months was captured by monthly random effects modeled by an autoregressive process of order 1. Models were adjusted for seasonality by including Fourier terms as a mixture of two cycles with periods of 6 and 12 months, respectively [26]. A yearly trend was fitted to estimate changes of the incidence rates over time. Bayesian variable selection implemented within the spatio-temporal model was applied to identify the most important ITN coverage indicator and lagged climatic factor with their functional form (i.e. linear or categorical). For the ITN indicators, a categorical variable was introduced into the model taking values 1–7, (six values corresponding to the six indicators and the seventh defined the absence of all indicators from the model). The probabilities of the above values were treated as parameters and used to estimate the likelihood of including the ITN indicator into the model, i.e. inclusion probability. Similarly, for each climatic factor, a categorical variable with three values was introduced corresponding to its absence, or inclusion into the model in linear or categorical form. The ITN indicator or climatic factor with the highest posterior inclusion probability was selected. Bayesian variable selection details are provided in Additional file 2.

Intervention and wealth score data from MIS, summarized at district level may not provide reliable estimates of the coverage because the survey is designed to produce reliable estimates at country and regional level. Therefore, coverage at district level was estimated by fitting Bayesian CAR binomial and Gaussian models on the aggregated intervention and wealth score data, respectively. Malaria cases seen at formal health facilities in Uganda are a fraction of the total cases due to low health-seeking behaviour [27], therefore, the models were adjusted for the proportion of malaria treatment-seeking behaviour reported in the most recent MIS survey [10]. However, the survey was designed to provide precise estimates of the malaria health seeking indicator at the country and regional level. Therefore, the Bayesian CAR model was used to obtain estimates at district-level [25]. District-level estimation modeling details are available in Additional file 3.

Models were implemented in OpenBUGS and parameters were estimated using Markov chain Monte Carlo (MCMC) simulation. A two-chain algorithm was run for 200,000 iterations with an initial burn-in period of 5000 iterations. Convergence was assessed by visual inspection of trace and density plots and analytically by the Gelman and Rubin diagnostic [28]. Parameters were summarized by their posterior medians and 95% Bayesian Credible Intervals (BCIs). Maps of estimated, smoothed incidence rates were produced in ESRI’s ArcGIS 10.2.1 (http://www.esri.com/). Details on model formulations are provided in the Additional file 4.

## Results

**Fig. 1:**
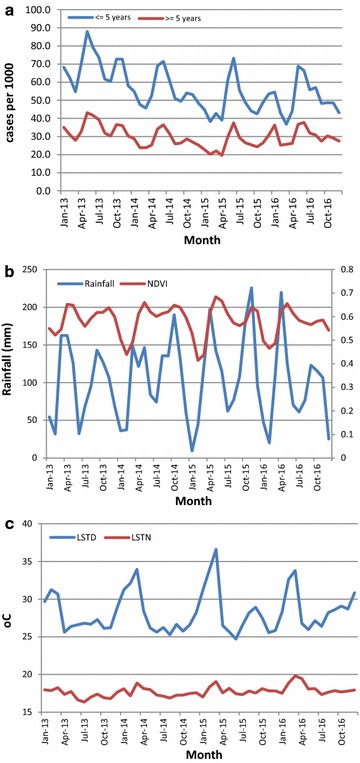
Temporal variation of monthly incidence and climatic factors during 2013–2016; **a** incidence, **b** Rainfall (primary axis) and NDVI (secondary axis), and **c** LSTD and LSTN

The annual number of malaria cases declined from 16,475,631 in 2013 to 13,724,255 in 2014 and to 13,057,293 in 2015, but rose to 15,016,031 in 2016, representing annual declines of 16.7 and 4.9%, and an increase of 15.0%, respectively. Malaria incidence in children < 5 years during the study period (i.e. Jan 2013–December 2016) was nearly two times higher than in individuals ≥ 5 years (Fig. 1). The average monthly incidence in children < 5 years declined steadily from 74.7 (95% CI 72.4–77.1) in 2013 to 49.4 (95% CI 42.9–55.8) in 2015, a decline of over 34% followed by an increase in 2016 of up to 51.3 (95% CI 42.9–55.8). In the older age group, a steady decline in monthly incidence from 2013 to 2015 was also followed by an increase in 2016.

The highest malaria incidence in children < 5 years was reported in Moroto district of North East region during December 2013 (334.5 per 1000 persons) and in older individuals, the highest incidence was observed in Ntungamo district in South western region during March 2016 (282.5 per 1000 persons). Temporal trends show a strong bi-annual seasonal pattern with two peaks during May–July and September–December (Fig. 1). The temporal variation of incidence in both age groups is highly positively correlated with that of climatic factors, but the extreme land surface temperature was negatively related to incidence.

Results from Bayesian variable selection of the ITN coverage indicators (Table 1) show that the proportion of population with access to an ITN in the household had the highest probability of inclusion among all ITN indicators. Therefore, this indicator was used as a measure of ITN coverage. Climatic averages of categorical forms of lags up to 2 months (LST, NDVI), and 3 months (rain) had higher inclusion probabilities in both age groups.

**Table 1:** Posterior inclusion probabilities for ITN coverage indicators

Indicator	Probability of inclusion	
	< 5 years (%)	≥ 5 years (%)
Proportion of households with at least one ITN	10.0	10.7
Proportion of households with at least one ITN for every two people	9.0	11.9
Proportion of population with access to an ITN in their household	56.2	48.5
Proportion of the population that slept under an ITN the previous night	2.5	12.5
Proportion of children under 5 years old who slept under an ITN the previous night	22.3	15.2
Proportion of existing ITNs used the previous night	0.0	1.2

Table 2 presents spatio-temporal estimates of the effects of interventions adjusted for climatic and socio-economic confounders. These results were obtained from models with only spatial random effects which provided a better fit to the data compared to models incorporating both spatial and non-spatial heterogeneities. For instance, the Deviance Information Criterion (DIC) was 83,370 and 83,579 for models on children < 5 years with only spatial and with both spatial and non-spatial random effects, respectively.

**Table 2:** Effects of interventions on malaria incidence estimated from Bayesian spatio-temporal models adjusted for socio-economic and climatic factors

Predictor	Children less than 5 years (n = 16,638,104)	Individuals 5 years and above (n = 41,345,996)
	IRR (95% BCI)	IRR (95% BCI)
Interventions^b^		
ITN	0.56 (0.41, 0.72)^a^	1.08 (1.00, 1.17)
ACT	0.72 (0.55, 0.89)^a^	0.75 (0.72, 0.80)^a^
Wealth index^c^		
Poorest (11,374,365)	1	1
Poorer (10,602,075)	0.87 (0.77, 0.98)^a^	0.88 (0.83, 1.93)
Middle (8,076,579)	0.77 (0.70, 0.84)^a^	0.80 (0.77, 0.84)^a^
Richer (12,828,925)	0.75 (0.71, 0.81)^a^	0.81 (0.73, 0.86)^a^
Richest (15,102,156)	0.79 (0.66, 0.97)^a^	0.84 (0.76, 0.95)
Proportion health seeking behavior	1.09 (1.07, 1.11)^a^	1.07 (1.04, 1.09)^a^
Rainfall (mm)		
≤ 76.9	1	1
77.0–125.7	1.02 (0.99, 1.05)	1.02 (0.95, 1.11)^a^
125.8–348.8	1.04 (1.01, 1.09)^a^	1.05 (1.01, 1.12)^a^
NDVI		
≤ 0.6	1	1
0.61–0.70	1.13 (1.09, 1.16)^a^	1.17 (1.14, 1.25)^a^
0.71–6.54	1.15 (1.12, 1.20)^a^	1.21 (1.17, 1.27)^a^
LSTD (°C)		
< 27.5	1	1
27.6–29.4	1.05 (1.02, 1.16)^a^	1.06 (1.02, 1.12)^a^
29.5–36.5	0.86 (0.80, 0.92)^a^	0.85 (0.82, 0.88)
LSTN (°C)		
< 18.0	1	1
18.1–18.5	0.99 (0.95, 1.02)^a^	0.97 (0.95, 1.05)
18.6–22.0	0.90 (0.86, 0.94)^a^	0.91 (0.89, 0.96)^a^
Altitude	0.80 (0.73, 0.88)^a^	0.92 (0.89, 0.94)^a^
% of district covered by crops	0.98 (0.91, 1.04)	1.00 (0.97, 1.02)
% of district covered by water	1.00 (0.95, 1.09)	1.00 (0.96, 1.04)

	Median (95% BCI)	Median (95% BCI)
Temporal trend		
2013	1	1
2014	0.002 (− 0.03, 0.02)	− 0.16 (− 0.19, − 0.14)
2015	− 0.13 (− 0.15, − 0.09)	− 0.06 (− 0.12, − 0.02)
2016	0.23 (0.19, 0.23)	− 0.12 (− 0.16, − 0.10)
Amplitude		
Annual	0.33 (0.15, 0.50)	0.28 (0.16, 0.78)
Semi-annual	0.11 (0.07, 0.20)	0.15 (0.09, 0.41)
Phase (months)		
Annual	2.66 (1.51, 5.68)	2.19 (1.40, 5.63)
Semi-annual	2.09 (1.16, 5.51)	1.56 (0.87, 4.99)
Spatial variance		
2013	1.20 (0.90, 1.57)	1.21 (0.91, 1.58)
2014	1.05 (0.79, 1.37)	1.00 (0.76, 1.30)
2015	1.52 (1.14, 1.99)	1.34 (1.01, 1.75)
2016	1.16 (0.87, 1.51)	1.04 (0.78, 1.36)
Temporal variance	16.89 (10.82, 25.05)	17.20 (11.06, 25.37)
Temporal correlation	0.94 (0.83, 0.99)	0.63 (0.10, 0.93)
Dispersion	14.03 (13.47, 14.60)	16.12 (15.49, 16.77)

^a^Statistically important effect

^b^Coverage was modeled on the scale of 0 to 1—therefore one unit increase in coverage corresponds to a 100% increase which implies a shift of the current by 100%

^c^Relative frequency distribution (a) < 5 years; poorest (22%), poorer (20%), Middle (13.4%), Richer (19%), Richest (25.6%) (b) ≤ 5 years; poorest (18.7%), poorer (17.6%), Middle (14.1%), Richer (23.4%), Richest (26.2%)

ITN coverage had a protective effect in children < 5 years but no statistically important effect in individuals ≥ 5 years. However, case management with ACT had a protective effect in both age groups. In particular, a 100% increase in the proportion of people sleeping under an ITN was associated with a decline in malaria incidence in children < 5 years of 44% (95% BCI 28–59%). A 100% increase in the proportion of fevers treated with ACT was related with a decline in incidence of 28% (95% BCI 11–45%) in children < 5 years and of 25% (95% BCI 20–28%) in older individuals. Socio-economic status was an important predictor of malaria incidence in both age groups, but the effect was much stronger in the younger group. The incidence is lower in the higher socio-economic levels.

The effects of environmental and climatic factors on malaria incidence were almost similar in the two age groups. In children < 5 years, incidence increased with higher rainfall, NDVI, and day LST, but decreased with altitude. However, excessive increase in LST was associated with a statistically important decrease in incidence. Similarly, for individuals ≥ 5 years, incidence increased with rainfall, NDVI, and LSTD, and decreased with altitude. Land cover had no effect on malaria incidence in both age groups.

Spatial variance in both age groups was highest in 2015 and lowest in 2014. In all years the spatial variability of incidence in young children was slightly higher than that of individuals ≥ 5 years except in 2013. However, temporal variation was much higher than spatial variability in all years. The temporal trend shows that malaria incidence in both age groups decreased during 2013–2015, and then increased again in 2016. The amplitude estimates suggest that malaria incidence was almost twice as high in children less than 5 years compared to older individuals. The seasonality phase parameters indicate that the peak of the malaria incidence occurs during February to May.

**Fig. 2:**
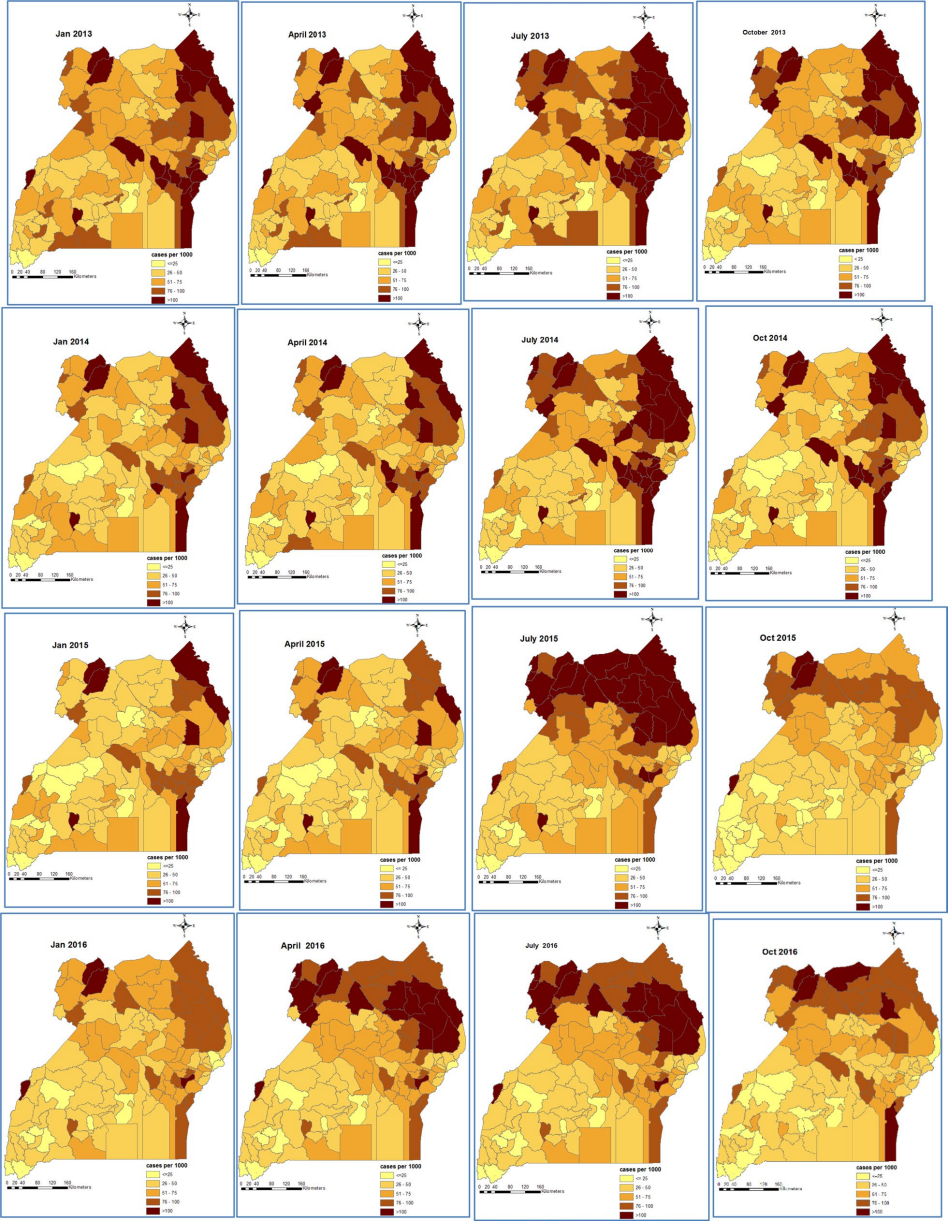
Space-time patterns of malaria incidence (cases per 1000 persons) in children less than 5 years estimated from the Bayesian spatio-temporal model

**Fig. 3:**
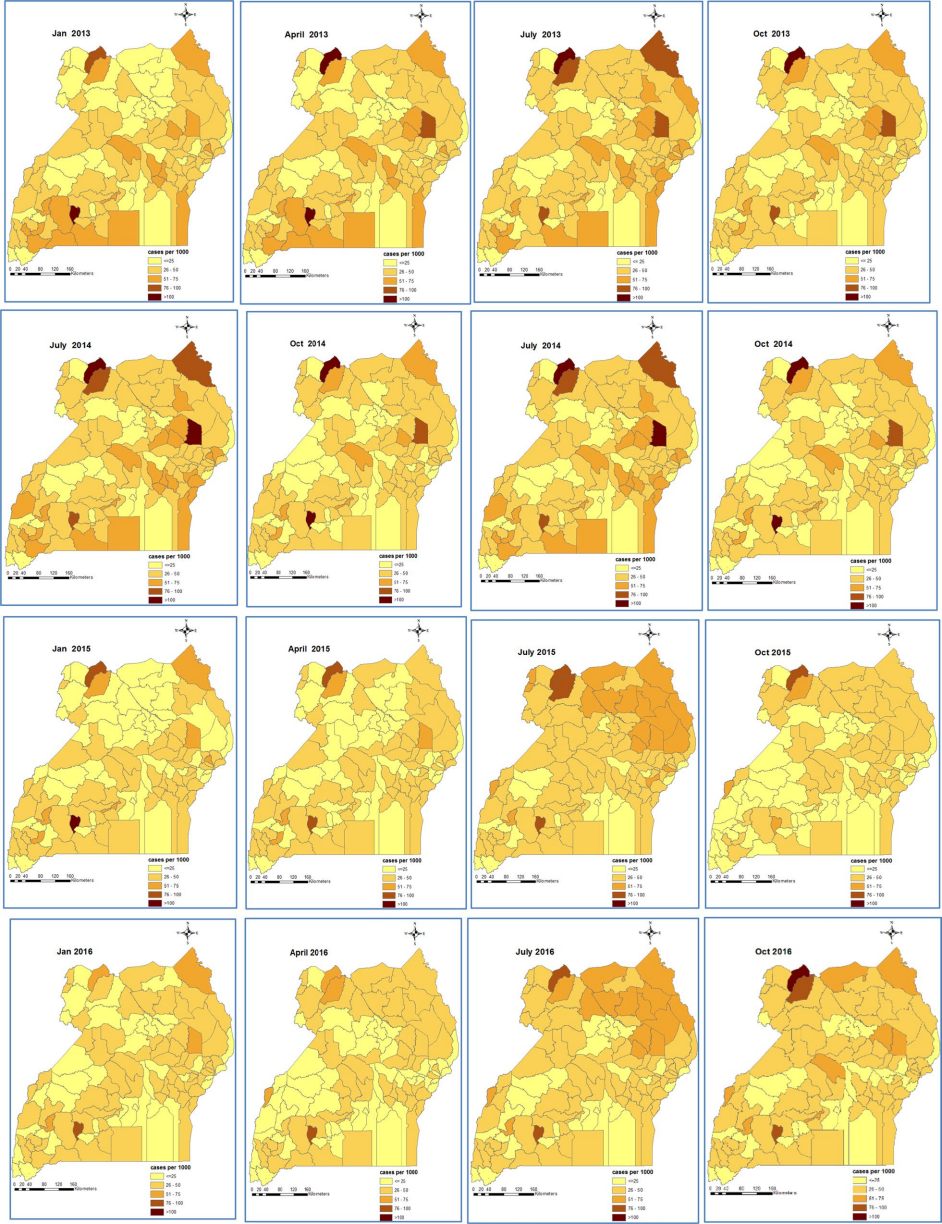
Space-time patterns of malaria incidence (cases per 1000 persons) in individuals of age 5 years and above estimated from the Bayesian spatio-temporal model

Maps of smoothed malaria incidence estimated from the Bayesian models are presented in Figs. 2 and 3 for the first month of each quarter and study year (i.e. January, April, July, and October). The space–time patterns of incidence differ between the two age groups. The high malaria burden districts throughout the study period were located in the Northern, North West and Eastern regions. In children < 5 years, the burden of malaria was high in 2013 with the majority of the districts having incidence rates of over 50 cases per 1000 persons. The districts located in the South western and Central regions had a much lower malaria incidence (< 25 cases per 1000 persons). In 2014, incidence rates declined except in the high burden districts of the North. Incidence declined further during the first and second quarters of 2015, reaching an overall district average of less than 25 cases per 1000 persons, and for the first time, the high burdened districts of North East had less than 75 cases per 1000 persons. However, starting from the third quarter of 2015 through 2016, an upsurge in incidence is apparent affecting mostly the North East region.

On the contrary, individuals ≥ 5 years had a much lower and homogeneously distributed burden throughout the country with small differences among districts. During 2013, incidence rates ranged between 25–50 cases per 1000 persons per month in most of the districts. A decline was observed through 2014 until the second quarter of 2015. Incidence started increasing at the beginning of the third quarter of 2015 up to the last quarter of 2016.

It is remarkable that the spatial patterns of malaria incidence in children < 5 years during October 2014–January 2015 bear a strong similarity with the predicted prevalence estimated from the MIS of 2014–15 which was conducted during the same period. They both indicate high burden in the regions of North East, West Nile, and East Central, and a very low burden in Kampala and South western regions.

## Discussion

In this study, the effects of ITN and case management with ACT on the space–time patterns of malaria incidence in Uganda were determined in the two age groups of below and above 5 years, using district-aggregated health facility data reported in the DHIS2 during January 2013–August 2016. Also, the smoothed space–time patterns of malaria incidence were estimated for all districts in the two age groups.

Results showed a decline in incidence between 2013 and 2014 followed by an increase in 2015. The temporal trends in the two age groups were characterized by a strong seasonal, bi-annual pattern with two peaks, at the end of the short (March–May) and longer (August–November) rainfall seasons, respectively. This result underlines the influence of rainfall patterns on inter and intra-annual variation of malaria burden in Uganda. The decline of malaria in children less than 5 years during 2013–2014 has been also shown in the analyses of the malaria indicator survey data of 2009 and 2014 [5].

A protective effect was estimated for ITN coverage in children less than 5 years and for ACT in both age groups. Unexpectedly, the ITN effect in older individuals was statistically not important, a result that may reflect that most ITN distribution campaigns are targeting children under 5 [6] and that young children have different sleeping patterns compared to adults. Young children tend to go to bed early and therefore are less exposed to mosquito bites if they sleep under an ITN, unlike adults who usually sleep late. However, some studies have reported no differences in ITN use between children and adults [29, 30]. The effectiveness of ITNs in young children derives from the endophagic nature of the *An. gambiae* vector, which feeds indoors where ITNs physically deter the vector from sucking a blood meal thus interrupting transmission between human and vector [31]. These findings agree with results reported from population surveys in Uganda [4, 5], and in other endemic settings [32].

Similarly, the effectiveness of ACT on malaria incidence in all ages derives from their action of suppressing and killing malaria parasites in the body, thus lowering the parasite load and consequently the chances of transmission [33]. The coverage and hence effectiveness of ACT has been further enhanced by the current national MoH guidelines that recommend the use of ACT and outlaw the use of other antimalarial drugs for malaria treatment in both private and public health facilities [6]. Similar findings have been reported in other studies [2, 5].

The space–time patterns of smoothed malaria incidence revealed heterogeneously distributed burden of high intensity in children under 5 years, but rather homogeneous spatial patterns of low intensity in older individuals. Young children have lower immunity which makes them highly susceptible to developing clinical malaria when they are bitten by infectious mosquitoes [34]. With the development of immunity in older individuals, the risk of clinical malaria decreases [35] and, therefore, geographical patterns of malaria incidence are more homogeneous.

The increase in malaria burden observed in 2015 may suggest changing malaria transmission dynamics as a result of sustained high intervention coverage which may lead to loss of immunity as a result of lower exposure to malaria [3]. Similar increases in incidence have been reported in other endemic countries where interventions have been scaled up in recent times including Zambia, Tanzania, and Rwanda [3]. The high burden of malaria incidence in the young children reported in the districts of North East, Eastern and West Nile regions could be attributed to differences in ecological conditions, and disparities in socio-economic development, urbanization, and access to health services [4, 5].

Study results further showed a protective effect of socio-economic status on clinical malaria in both age groups which is stronger however in children under 5 years. Socio-economic status is a key confounder for epidemiological outcomes and it is the most important determinant of health in young children [36]. The effect of socio-economic status on malaria incidence is also reflected on the spatial patterns of the disease that revealed a lower burden in affluent districts such as Kampala and Wakiso, but a high burden in the socioeconomically disadvantaged districts of Moroto, Kotido and Nakapiripirit in the North East. This finding confirms existing knowledge that higher socio-economic regions have a much smaller malaria burden compared to poverty-stricken ones [37].

Rainfall, Normalized Difference Vegetation Index, day and night land surface temperature, and attitude were significantly associated with malaria incidence in both age groups. Land surface temperature influences the survival of the mosquito vector and the duration of development of the vector and the parasite [38]. The reduced risk of incidence associated with extreme day land surface temperature is due to reduced mosquito survival at high temperatures [23, 24, 39]. These results are in agreement with findings from other studies that employed spatio-temporal analyses of routine health facility malaria data in Zimbabwe [17] and in Yunan Province, China [18], but slightly differ with results reported from a study in northern Malawi [16]. Non-spatially structured heterogeneity was much higher than the spatially structured variability, which may imply high endemicity across the country irrespective of the geographical location. The temporal variation was higher than the spatial one in both age groups reflecting the stronger influence of seasonality in malaria transmission which is linked to climatic variability. The close relationship between malaria and climatic factors could be exploited to develop a malaria early warning system for predicting malaria outbreaks [40]. Similar findings were the basis for the development of forecasting models in Burundi [41], Ethiopia [23] and Botswana [42]. It is interesting however to note that the seasonal pattern in malaria incidence varied across the country supporting the evidence of a complex relationship between climatic factors and malaria transmission and the need for regionally adapted forecasting models.

The space–time patterns of malaria incidence in children < 5 are similar to those of parasitaemia prevalence predicted from the MIS 2014–15 [4]. This is an indication of the improved quality of routinely collected health facility data that can be attributed to the benefits of the DHIS2 implementation in Uganda [8].

A major limitation of the current study is the use of CAR models which are prone to estimation biases due to the ecological fallacy [34]. This means that outcome-exposure relationships at the individual level may be different at an aggregated level. On the other hand, point process models such as log-Gaussian Cox model [43], produce precise parameter estimates, but their application requires analysis of case locations data which is not available in the Uganda DHIS2 system. The data is instead reported in aggregate form at the catchment area of the health facility. However, the MoH has started piloting an electronic data record system—Open Medical Records Systems (OpenMRS)—with a plan to replace the current paper data collection by early 2019 [44]. Once the roll-out is completed, individual case data will be available including locations which will enable us to repeat the analyses using point process models. The models will be fitted using the Integrated Nested Laplace Approximation (INLA) approach owing to the complexity of computations involved that would otherwise make MCMC infeasible.

## Conclusions

The decline in malaria incidence during 2013–2015 highlights the success of vector-control interventions and case management with ACT on the fight against malaria in Uganda. This calls for sustaining these prevention efforts to achieve universal coverage and curb the reverses in malaria decline observed in 2016. NMCP should speed up the scale-up of indoor residual spraying of households in the districts of North East and Eastern regions to reduce the persistently high burden of disease. The close similarity of disease patterns obtained in this study to the population-based survey estimates highlight the relevance of routinely collected data in disease burden estimation.

## Additional files

**Additional file 1.**Climatic data processing.

**Additional file 2.** Bayesian variable selection.

**Additional file 3.** Estimating district-level indicator estimates.

**Additional file 4.** Statistical modeling details.

## Abbreviations

DHIS2: District Health Information Software System version 2
ACTs: artemisinin-based combination therapy
CAR: conditional autoregressive
MIS: Malaria Indicator Surveys
ITNs: insecticide-treated nets
IRS: indoor residual spraying
RDTs: rapid diagnostic tests
RBM: Roll Back Malaria
WHO: World Health Organization
UMRSP: Uganda Malaria Reduction Strategic Plan
SSA: Sub-Saharan Africa
MoH: Ministry of Health
NMCP: National Malaria Control Programme
RS: remote sensing
LST: land surface temperature
NDVI: Normalized Difference Vegetation Index
MODIS: Moderate Resolution Imaging Spectroradiometer
BCI: Bayesian credible intervals
DHS: Demographic health survey
