# Supplementary material for: The effect of case management and vector-control interventions on space–time patterns of malaria incidence in Uganda
Source: Malar J. 2018 Apr 12;17:162. doi: 10.1186/s12936-018-2312-7 (PMC5898071; doi:10.1186/s12936-018-2312-7)
Supplement: Supplementary file 1 — Additional file 1.Climatic data processing. [file 12936_2018_2312_MOESM1_ESM.docx]

**Climatic data processing**

The climatic data downloaded from MODIS that is, LSTD, LSTN and NDVI were available in the .hdf format - a raster data format. Data for each climatic factor and period was stored in different "granules", which are tile shaped squares formed by borders of intersecting latitudes and longitudes on the earth surface. Uganda is covered by 4 such granules bounded by decimal latitude and longitude borders of N(4.234077), E(35.00000), S(-1.478794), and W(29.572774).Data for each climatic factor at a single period/time point consisted of 4.hdf files. The .hdf files were converted into other formats prior to extracting the values of each climatic factor for every district centroid using python scripts created by authors in ArcGIS. The conversions were carried out; i) combining granules to a single .hdf file, ii) conversion from .hdf file to. tiff file, iii)conversion from .tiff to ASCII format that can be read in statistical software such as STATA and R.

The dekadal rainfall data was available the .bil files format from the US early warning and environmental monitoring system. The .bil files formats were converted directly into ASCII files using customised python scripts.

For each district, monthly climatic factor estimates of LSTD, LSTN and NDVI were calculated using average function at the centroid. For rainfall it was the cumulative values that gave the total rainfall in the month.

The data was then reshaped from wide to long format, merged with malaria cases of a specific month and year belonging to a given district. Finally, three month lags were created for climatic data.
