# Supplementary material for: The effect of case management and vector-control interventions on space–time patterns of malaria incidence in Uganda
Source: Malar J. 2018 Apr 12;17:162. doi: 10.1186/s12936-018-2312-7 (PMC5898071; doi:10.1186/s12936-018-2312-7)
Supplement: Supplementary file 2 — Additional file 2. Bayesian variable selection. [file 12936_2018_2312_MOESM2_ESM.docx]

**Bayesian variable selection**

To choose the most important ITN coverage indicator that explains the maximum variation in malaria incidence, Bayesian variable selection using stochastic search was implemented separately for ITN indicators, and environmental and climatic factors. For ITN indicators, a categorical variable $X_{p}$ was introduced into the model and assigned values 1 to 7 representing exclusion of the variable from the model$\left( I_{p}=1 \right)$, and inclusion of the six indicators as follows; proportion of existing ITNs used the previous night $\left( I_{p}=2 \right)$, proportion of children under five years old who slept under an ITN the previous night $\left( I_{p}=3 \right)$, proportion of the population that slept under an ITN the previous night $\left( I_{p}=4 \right)$, proportion of households with at least one ITN for every two people $\left( I_{p}=5 \right)$, proportion of households with at least one ITN $\left( I_{p}=6 \right)$, and proportion of population with access to an ITN in their household $\left( I_{p}=7 \right)$. Also, for lagged climatic predictors, a categorical variable $Y_{p}$ was created with values 1 to 7 introduced into the model to represent exclusion of the variable from the model$\left( I_{p}=1 \right)$, and inclusion of different variables as follows; lag1 (continuous) $\left( I_{p}=2 \right)$, lag1 (categorical) $\left( I_{p}=3 \right)$, lag2 (continuous) $\left( I_{p}=4 \right)$, lag2 (categorical) $\left( I_{p}=5 \right)$, lag3 (continuous) $\left( I_{p}=6 \right)$ and lag3 (categorical) $\left( I_{p}=7 \right)$ For non-lagged climatic factors that is, altitude and distance to water bodies, a categorical variable $Z_{p}$ with three values was defined representing exclusion from model $\left( I_{p}=1 \right)$, inclusion of continuous form $\left( I_{p}=1 \right)$, and inclusion of categorical form $\left( I_{p}=2 \right)$. In the latter scenario, $I_{p}$ has a probability mass function $\prod_{j=1}^{2} \pi_{j}^{\delta_{j}\left( I_{p} \right)}$, where $\pi_{j}$ denotes the inclusion probabilities of functional form j (j=1,2,3) so that $\sum_{j=1}^{3} \pi_{j}=1$ and $\delta_{j}\left( . \right)$ is the Dirac function, $\delta_{j}\left( I_{p} \right)=\left\{ \begin{aligned} 1, &if I_{p}=j \\ 0, &if I_{p} \neq j \end{aligned} \right. .$A spike and slab prior distribution was assumed for the regression coefficients. In particular for the coefficient $\beta_{p}$ of the corresponding variable X_p_, we assumed $\beta_{p}\sim\delta_{1}\left( I_{p} \right)N\left( 0,\tau_{p}^{2} \right)+\left( 1-\delta_{1}\left( I_{p} \right) \right)N\left( 0,\vartheta_{0}\tau_{p}^{2} \right)$, that is a non-informative prior for $\beta_{p}$ if $X_{p}$ is included in the model (slab) and an informative normal prior shrinking $\beta_{p}$ to zero (spike) if $X_{p}$ is excluded from the model, setting $\vartheta_{0}$to be a large number, e.g, 10^5^. Similarly, $\beta_{p,l}\sim\delta_{2}\left( I_{p} \right)N\left( 0,\tau_{p,l}^{2} \right)+\left( 1-\delta_{2} \right)N(0,\vartheta_{0}\tau_{p,l}^{2})$ was assumed for the scenario of selecting one out of six indicators/variables or exclusion of the variable. The coefficients {$\left. \beta_{p,l} \right\}_{l=1,..,7}$ corresponding to inclusion of $X_{p}$, p=1,…,7 in the model. For inclusion probabilities, a non-informative Dirichlet distribution was adopted with hyper parameter$\alpha={(1,1,1,1,1,1,1)}^{T}$, that is,$\boldsymbol{\pi}={(\pi_{1},\pi_{2},\pi_{3},\pi_{4},\pi_{5},\pi_{6},\pi_{7})}^{T}\sim Dirichlet\left( 7,\alpha\right).$ We also assumed inverse Gamma priors for the precision hyper parameters $\tau_{p}^{2}$ and $\tau_{p,l}^{2}$, $l=1,\ldots,7$.
