# Supplementary material for: The effect of case management and vector-control interventions on space–time patterns of malaria incidence in Uganda
Source: Malar J. 2018 Apr 12;17:162. doi: 10.1186/s12936-018-2312-7 (PMC5898071; doi:10.1186/s12936-018-2312-7)
Supplement: Supplementary file 3 — Additional file 3. Estimating district-level indicator estimates. [file 12936_2018_2312_MOESM3_ESM.docx]

**Estimating district-level interventions coverage, socioeconomic status, and health seeking behavior**

Data for intervention coverage, wealth index and health seeking behavior were only available at regional level from the MIS 2014-15 and DHS 2016 surveys. This is because the population based surveys are designed to give precise estimates only at regional and country levels. A Conditional Autoregressive (CAR) model was developed to estimate district level estimates of formulated with a binomial distribution for intervention coverage and health seeking behavior indicators, and a Gaussian distribution for the wealth score, a measure of socioeconomic status. Slightly fewer than all the 112 districts had clusters selected in the original sample, therefore to fit the CAR models the districts with missing data were assigned a median value of the districts located within a specific region. The models were formulated as follows;

Let $Y_{i}$ be the number of households that possessed at least one ITN in district $i=1,\ldots,112$, and $N_{i},$ the total number of households sampled and interviewed in district $i$. We assume that $Y_{i}$ follows a Binomial distribution, that is, $Y_{i}|N_{i},\pi(i)\sim Bin(N_{i},\pi(i))$ $\forall i=1,\ldots,112$, where $\pi(i)$ is the proportion of households with at least one ITN in district i. A Bayesian CAR model to estimate district-level ITN coverage was formulated as follows;

$\mathrm{logit}\left( \pi(i) \right)=\beta_{0}+\omega_{i}$, where $\beta_{0}$ is a constant, and $\omega_{i}$, *i*=1,…,112, are modeled via a CAR process. Each $\omega_{i}$ conditional on the neighbor $\omega_{j}$ follows a normal distribution with mean equal to the average of neighboring districts $\omega_{j}$ and variance inversely proportional to the number of neighbor districts$n_{i}$, that is; $\omega_{i}|\omega_{j}\sim N\left( \gamma\sum_{l\in\delta_{i}} \omega_{j},\frac{\sigma_{\omega}^{2}}{n_{i}} \right)$, where γ quantifies the amount of spatial correlation present in the data, $\sigma_{\omega}^{2}$ measures the spatial variance. $\omega_{i}$ and $\omega_{j}$ are adjacent districts in the set of all adjacent districts $\delta_{i}$ of district $i$, and $n_{i}$ are the number of adjacent districts. Following standard formulation of Bayesian regression models, we assumed vague priors; A non-informative Gaussian distributions with mean 0 and variance 10^2^ for $\beta_{0}$, that is, $\beta_{0}$~N(0, 10^2^). An inverse gamma prior distribution with mean 10 and variance 100 was considered for $\sigma_{\omega}^{2}$, i.e. $\sigma_{\omega}^{2}\sim Ga\left( 0.1,0.001 \right).$

Similar formulations were applied for ACTs, malaria treatment seeking behavior, and household asset index, however the latter was modeled by a first stage Gaussian distribution.
