# Supplementary material for: The effect of case management and vector-control interventions on space–time patterns of malaria incidence in Uganda
Source: Malar J. 2018 Apr 12;17:162. doi: 10.1186/s12936-018-2312-7 (PMC5898071; doi:10.1186/s12936-018-2312-7)
Supplement: Supplementary file 4 — Additional file 4. Statistical modeling details. [file 12936_2018_2312_MOESM4_ESM.docx]

**Bayesian model formulation**

Let $Y_{it}$ be the number of malaria cases reported in calendar month *t=1,…,12,*  year *j=1,…,4* and district $i=1,\ldots,112$. $Y_{ijt}$ is assumed to follow a negative binomial distribution, $Y_{ijt}\sim NB\left( p_{ijt}, r \right)$ where $p_{ijt}=\frac{r}{r+\mu_{ijt}}$ where $r$ is the dispersion parameter and $\mu_{ijt}$ is the average number of monthly malaria cases in the district. The model is formulated with a log link function that is; $\log\left( \mu_{ijt} \right)=\log\left( N_{ijt} \right)+\alpha+X^{T}\beta+f_{T}\left( Z_{j} \right)+f_{s}\left( t \right)+\omega_{ij}+\theta_{ij}+\epsilon_{\left( j-1 \right)*12+t}$ if both spatial and non-spatial random effects are incorporated, or

$\log\left( \mu_{ijt} \right)=\log\left( N_{ijt} \right)+\alpha+X^{T}\beta+f_{T}\left( Z_{j} \right)+f_{s}\left( t \right)+\omega_{ij}+\epsilon_{\left( j-1 \right)*12+t}$, if only spatial random effects are assumed.

Where $N_{ijt}$ is the offset district-month specific population, α is the intercept, $\beta$ is a vector of regression coefficients associated with the vector of predictors $X_{it}$ (interventions, environmental, socio-economic status). $\epsilon_{\left( j-1 \right)*12+t}$ are monthly random effects modeled by a first order autoregressive process with temporal variance $\sigma_{1}^{2}$, that is, $\epsilon_{l}\sim AR\left( 1 \right)$ where $\epsilon_{1}\sim N\left( 0,\frac{\sigma^{2}}{1-\rho^{2}} \right)$, $\epsilon_{l}\sim N\left( \rho\epsilon_{l-1},\sigma^{2} \right), l=2,\ldots,43$ and the autocorrelation parameter $\rho$

quantifies the degree of dependence between successive months.$f_{T}\left( Z_{j} \right)$and $f_{s}\left( t \right)$ are parameters modeling the time trend and seasonality, $f_{T}\left( Z_{j} \right)$ describes an annual trend with the year $Z$ treated as categorical covariate$\omega_{i}$ is the spatial random effect for district *i* . The seasonal pattern $f_{s}\left( t \right)$ was captured by a mixture of two harmonic cycles with periods $T_{1}=$6 and $T_{1}=12$ months, respectively, that is, $f_{s}\left( t \right)=\sum_{j=1}^{2} A_{j}\cos\left( \frac{2\pi}{T_{j}}t-\varphi_{j} \right)=\sum_{j=1}^{2} \{a_{j}*cos\left( \frac{2\pi}{T_{j}}t \right)+b_{j}*sin(\frac{2\pi}{T_{j}}t)\}$, where $t$ is time in months. $A_{j}$ is the amplitude of the $jth$cycle and estimates the incidence peak by the expression $A_{j}=\sqrt{a_{j}^{2}+b_{j}^{2}}$. $\varphi_{j}$is the phase which is the point where the peak occurs estimated as $\varphi_{j}=\arctan\left( \frac{a_{j}}{b_{j}} \right)$, $a_{j}$ and $b_{j}$ are model parameters. The $\omega_{ij}$ are district- year specific random effects, modeled via conditional autoregressive CAR($\sigma_{1j}^{2}$) processes. Each $\omega_{ij}$ conditional on the neighbor $\omega_{kj}$ follows a normal distribution with mean equal to the average of neighboring districts $\omega_{kj}$ _,_$k\in\delta_{i}$ and variance inversely proportional to the number of neighbor districts $n_{i}$, that is; $\omega_{ij}|\omega_{kj}\sim N\left( \gamma_{j}\sum_{k\in\delta_{i}} \omega_{kj},\frac{\sigma_{2j}^{2}}{n_{i}} \right)$, where $\gamma_{j}$ quantifies the amount of spatial correlation present in the data in year $j$, $\sigma_{2j}^{2}$ measures the spatial variance. $\omega_{ij}$ and $\omega_{kj}$ are adjacent districts in the set of all adjacent districts $\delta_{i}$ of district $i$, and $n_{i}$ are the number of adjacent districts. $\theta_{ij}$are exchangeable district-year random effects, i.e. $\theta_{ij}\sim N(0,\sigma_{2j}^{2})$.

A non-informative normal prior distribution was assumed for the regression coefficients, a Gamma distribution with mean 1 and variance 100 for the parameter, r, an inverse gamma prior distribution with mean 10 and variance 100, for ${\sigma_{1j}^{2}, \sigma}_{2j}^{2},\sigma^{2}$ and $\sigma^{2}$, i.e. ${\sigma_{1j}^{2}, \sigma}_{2j}^{2},\sigma^{2}\sim Ga\left( 0.1,0.001 \right), j=1,\ldots4$ and a Uniform prior distribution for $\rho$, i.e. $\rho\sim U\left[ -1,1 \right]$.
